# Optimal surgery for resectable malignant pleural mesothelioma in the setting of multimodality treatment

**DOI:** 10.1007/s00595-023-02723-8

**Published:** 2023-07-20

**Authors:** Nobuyuki Kondo, Seiki Hasegawa

**Affiliations:** Department of Thoracic Surgery, Hyogo Medical University, 1-1 Mukogawa-Cho, Nishinomiya, Hyogo 663-8501 Japan

**Keywords:** Malignant pleural mesothelioma, Surgery, Multimodality treatment, Macroscopic complete resection

## Abstract

The surgical treatment of malignant pleural mesothelioma (MPM) involves procedures to achieve macroscopic complete resection, depending on the patient’s condition. We reviewed the evolution of surgical approaches for resectable MPM. Since surgery is no more than a single step in the set of processes in multimodality treatment (MMT), we concluded that these procedures should give precedence to lung preservation and minimize resection whenever possible. Postoperative quality of life must be prioritized when the patient can receive appropriate adjuvant therapy.

## Introduction

Radical surgery for resectable malignant pleural mesothelioma (MPM) aims to achieve macroscopic complete resection (MCR) [1, 2]. Conversely, combining chemo-, radiation, and novel therapies has improved the prognosis of patients with MPM [3–5]. The treatment outcomes of patients with resectable MPM, including overall survival (OS), are improved in a multimodality treatment (MMT) setting. Recent guidelines recommend the MMT protocol combined with various modalities, including surgical therapy, based on the results of trials. However, there is still no final consensus on the standard treatment sequence [6, 7]. There are two surgical procedures to treat MPM: extra-pleural pneumonectomy (EPP) and pleurectomy/decortication (P/D) to preserve the lung parenchyma [8]. At the 2011 international mesothelioma interest group (IMIG) annual meeting, the debate on “whether EPP or P/D should be done” for resectable MPMs reached the consensus that “the type of cytoreductive procedure should be selected based on the disease distribution, institutional experience, and the surgeon’s preference and experience” [2]. Both procedures can achieve MCR, but P/D presents several advantages over EPP in sparing patients’ lungs, reducing postoperative complications, and improving their quality of life (QoL) [9]. Therefore, P/D is the surgical procedure of choice for MPM at major facilities (high-volume centers) in Europe and the United States [10, 11].

Surgical techniques have gained stability in line with the increasing number of MPM cases in experienced institutions [8, 12–16]. At the same time, MMT improves the survival rate. In addition to the standardization of pemetrexed-platinum combination therapies, some promising new therapies established in the treatment of lung cancer, such as angiogenesis inhibitors, immune checkpoint inhibitors, and targeted therapies, have been introduced into the treatment of MPM [3–5, 17, 18]. Incorporating the benefits of these new therapies into postoperative adjuvant therapy and post-recurrence treatment may improve long-term prognosis. Thus, it is essential to consider postoperative cardiopulmonary function and QoL when choosing a surgical procedure [19]. This review aims to identify the optimal surgical procedure for improving the outcomes of MPM treatment.

### Definition of surgical treatment for MPM

Surgery for MPM, which is mainly cytoreductive, considers the characteristics of pleural tumors; however, due to the histological nature of MPM, microscopic residual tumor cells in the surgical margin cannot be avoided [20]. In 2006, Sugarbaker et al. defined the goal of surgical treatment for MPM as a macroscopic complete resection (MCR) [1].

Theoretically, based on the definition of R0, R1, and R2 resections, MCR stands for R1 surgery considered to be the maximum radicality possible for MPM [21]. At the 2019 International Association for the Study of Lung Cancer taskforce, Friedberg et al. defined the specific method of achieving MCR as follows: “no visible residual tumor or palpable cancer, equal to R1 resection” [22]. Moreover, the following consensus on surgical treatment was reached at the 2012 IMIG annual meeting in Boston: “in a surgical indication where MPM is histologically diagnosed, there is a lesion for which MCR can be achieved, and other therapeutic means can be implemented” [2]. Considering the importance of surgical treatment, we must mention the Mesothelioma and Radical Surgery (MARS) trial, the only randomized phase 3 clinical trial that involves surgical treatment [23]. However, some evaluations of the MARS trial reported that the number of patients is statistically underpowered (EPP (*n* = 24), no EPP (*n* = 26)) and the conclusion is too bloated outside the setting of the primary endpoint [24, 25]. Consequently, negative assessments for surgical treatment presented as outcomes must be managed carefully. Subsequently, the MARS researchers have stated that careful evaluation is needed [26].

### Trend of surgical procedures

Between the 1970s and 2000s, curative-intent surgery for MPM indicated EPP followed by P/D [27, 28]. Conversely, the current guideline recommends the choice of P/D as lung-sparing surgery (ERS/ESTS/EACTS/ESTRO guidelines for the management of MPM, 2020) [6].

Looking back at the early MPM surgical interventions, there was only pleurectomy, which was performed as palliation [29]. Subsequently, two types of curative-intent surgery were reported: EPP and lung-sparing surgery, the so-called P/D. EPP was initiated in the 1970s [27] and P/D was reported several years later as less invasive with a lower significant morbidity rate [30]. Technically, the first half of the procedure is common and consists of the parietal pleura detachment [31]. In 2012, at their annual meeting in Boston, IMIG members discussed whether to use EPP or P/D for resectable MPM. They agreed that the surgeon should select the appropriate surgical method for each case, as long as MCR could be achieved [2].

Comparing postoperative mortality and adverse events, EPP had significantly higher mortality and complication rates than P/D (6.8% vs. 2.9% and 62.0% vs. 27.9%, respectively) [4]. On the other hand, P/D may be less curative oncologically than EPP [32]. In this context, no clinical trial has directly compared EPP and P/D and will be impossible to conduct in future. For this reason, there is no clear evidence of the superiority or inferiority of either of these procedures [33]. Since both EPP and P/D are designed for R1 resections, their radicality of tumor resection and oncological significance are considered equivalent. As P/D has relatively lower rates of severe complications and the advantage of being lung-sparing [21], EPP is not prioritized over P/D. Since 2008, evidence of clinical practices showed that surgeons who chose P/D had better results than those who performed EPP. The median survival rates (months) are reported as: 10.4–26 vs. 6.0–19.5 [10], 16 vs. 12 [12], 17 vs. 13 [34], 23 vs. 12.8 [35]. Moreover, because perioperative complications are reduced with P/D (27.9% (P/D) vs. 62.0% (EPP), *P* < 0.01) [4], postoperative QoL is not compromised, and postoperative treatment and retreatment at the time of recurrence are possible, the surgical method that preserves the lungs is considered superior [10, 25, 35, 36].

Since 2012, we have modified resection for MPM to prioritize P/D at our hospital [36]. Based on the findings during surgery, the surgeon can decide whether to resect the diaphragm or the pericardium. In a multicenter clinical trial in Japan, we tried to unify the surgical techniques specified in the document for quality control of P/D [37]. Following the 8th edition of the TNM staging system, we revised the surgical indications. Furthermore, we recently introduced non-incisional P/D in the expectation that the prognosis of resected MPM patients would be further improved [38].

Here we introduce Japan's first phase II multicenter clinical trial (JMIG0601), a clinical study that examined the validity (feasibility study) of a tri-modality strategy (induction chemotherapy, EPP, and radiation) for resectable MPM, conducted between May 2008 and November 2010. The primary endpoints of MCR > 70% and mortality < 1.0% were achieved. However, the results were unsatisfactory, with a median survival of 19.9 months and treatment-related mortality of 9.5% [39].

Following the lead of Europe and the U.S., which switched to P/D in about 2000, a second phase II multicenter clinical trial was conducted in Japan between November, 2012 and October, 2013, demonstrating the validity of P/D in multimodality treatment. The primary endpoint of MCR achievement was > 90% and the 30-day/90-day mortality rates were 0%. Median survival was 43.3 months, surpassing previous results, and the ratio of pre- and postoperative lung function was FVC/FEV1.0 = 78%/82.5% during the postoperative follow-up period. The results of this study led to the switch to P/D in Japan [37].

### Surgical treatment is indicated only when performed as part of MMT

Theoretically, surgical resection for MPM focuses on R1 resection, where surgery is only included as part of combined therapy. However, there is evidence that MMT contributes to OS more than treatment with a single modality. A retrospective analysis of the IASLC database (*n* = 1360) showed a median survival of 20 months for the multidisciplinary treatment group vs. 11 months for the surgery alone group [40]. Although the European and United States guidelines recommend MMT, no standard treatment sequence has been established until now.

In the 1970s, MPM treatment required adjuvant therapy either before or after surgery because the surgical resection leaves inevitable microscopic tumor cells [27]. Therefore, the results of surgery alone for MPM are poor, with a median survival time (MST) of 11–13 months vs. > 20 months achieved by MST combined with adjuvant therapy. Therefore, MMT contributes to a better prognosis [40, 41]. As such, surgical resection should be performed as part of MMT to improve outcomes [42]. It is ideal to “perform treatment based on an appropriate protocol” [24], but the optimal treatment sequence for chemotherapy and surgery has not been established. A prospective controlled trial of preoperative and postoperative chemotherapy (EORTC 1205) has been conducted in in Europe since 2018. The results of this trial are expected to give the first answer to the previously debatable question [43]. The consensus and guidelines of several academic societies recommend MMT for resectable MPM. In this context, the following guidelines recommend including surgery as part of MMT: ERS and ETS in 2010 and ERS/ETS/EACTS/ESTRO guidelines in 2020 [6, 7, 44]. On the other hand, some facilities use a combination of intraoperative treatment to enhance local control, but no related guideline recommendations exist [45]. There are also reports on the effectiveness of other treatments as local controls, such as hyperthermic intraoperative cisplatin chemotherapy [46] and photodynamic therapy (PDT) [47].

### Postoperative QoL

The effects of immune checkpoint inhibitors and novel treatments in systemic cancer therapy have been observed [17]. Furthermore, the combination of chemo-, immuno-, and targeted therapies has evolved, with further improvements in cancer treatment results expected. Following the improvement in prognosis, the next focus is on postoperative QoL. If the latter is preserved adequately, we may be able to expand treatment indications for recurrence. For this reason, QoL has become essential in determining the selection and adaptation of surgical procedures [48]. Therefore, even for MPM, P/D, which maintains a high QoL and expands the options for additional treatment, tends to be more advantageous than EPP. A comparison of 659 cases (102 EPP, 432 P/D) extracted in a systematic review found that postoperative QoL tended to be better after P/D [49]. A single-center study reported that after EPP, the median vital capacity decreased significantly from 2.8 L (77.7%) to 1.8 L (47.6%) [50]. Adjuvant therapy and post-relapse treatment are indicated for many patients. In our single institution study, among 57 patients with recurrence after P/D (1-year post-recurrence survival rate, 59.5%) and 39 with recurrence after EPP (1-year post-recurrence survival rate, 40.0%), 43 (75.4%) of the P/D group and only 21 (53.8%) of the EPP group underwent post-recurrence treatment [19, 51]. The contribution of P/D surgery on QoL may also be based on an ongoing comparative study between surgery plus chemotherapy and chemotherapy alone (MARS2 trial) [52].

### Has the role of EPP ended?

EPP was first reported by Butchart and is well established as a curative surgery for MPM [27]. The first half of the procedure, parietal pleurectomy, is common for EPP and P/D [31]. In considering which surgical technique to use, we summarized reports from several high-volume centers comparing EPP and P/D in multimodality treatment (Table 1) [8, 10, 12–16, 36, 53–55]. The superiority and inferiority of EPP vs. P/D have been reported from various viewpoints. According to one meta-analysis, the MST was about 12–20 months for EPP and 7.1–31.7 months for P/D in a systematic review, being slightly better for P/D; however, the data was insufficient [4]. EPP is more surgically invasive and has far more complications, whereas adjuvant radiotherapy is possible only after unilateral pneumonectomy [56]. Some post-EPP retrospective analyses reported good OS for epithelioid MPM patients without lymph node metastases [57, 58].

**Table 1:** Comparison of the results of extra-pleural pneumonectomy and pleurectomy/decortication as part of multimodality treatment for resectable malignant pleural mesothelioma

Author	*N* of Pts	EPP/P/D	Period	Age	Laterality right (%)	MMT completion (%)	Stage I–II (%)	Histology epithelioid (%)	Overall survival Median (mo), 2 y (%), 5 y (%)	DFS (months)	Recurrence rate (%)	Mortality (%) 30-day 90-day	(> Grade 3) complication (%)	Study design	MCR
		EPP/P/D		EPP/P/D	EPP/P/D	EPP/P/D	EPP/P/D	EPP/P/D	EPP/P/D	EPP/P/D	EPP/P/D	EPP/P/D	EPP / P/D		
Flores [12]	663	385/278	1990–2006	60/63	56/62	69/58	25 (96)/35 (98)	69/64	12/16, NA/NA	NA	local 33, distant 66/ local 65, distant 35	7.0/4.7 NA	NA	Retro	NA
Luckraz [10]	139	49/90	1995–2009	55.4/60.8	65.6/66.5	75.5/62.2	stage I 65.3/12.5	46.9/51.6	19.5/26.0	NA	NA	8.1/1.1 NA	41.2/15.8	Retro	NA
Burt [13]	225	95/130	2009–2011	63.2/68.3	NA	NA	NA	NA	NA	NA	NA	10.5/3.1 NA	24.2/3.8	Retro	NA
Bovolato [53]	503	301/202	1982–2012	58.7/62.5	NA	68.4/78.7	c: 66.8/64.7 p: 24.6/50	86.6/77	18.8/20.5 2y: 37/40 5y: 12/10	NA	NA	4.1/2.6 6.9/6	21.6/10.4	Retro	NA
Batirel [14]	130	42/66	2003–2014	55.7	NA	NA	48 (EPP+P/D)	75	18.3/14.6 2y: 35/35	NA	NA	7/2 NA	NA	Retro	NA
Sharkey [15]	362	133/229	1999–2014	57/65	NA	NA	p: 14.3/20.1	72.2/75.5	12.9/12.3 NA	11.5/10.6	85.0/79.0	6.0/3.5 13.5/9.2	88.7/93	Retro	NA
Kostron [54]	167	141/26	1999–2015	61/66	55/69	100/100	p: 29/35	64/94	23/32 NA	15/13	NA	5/0 10/0	93/77	Retro PSM	NA
Verma [55]	1307	271/1036	2004–2012	65/69	NA	76/61	c: 48/53	34/26	19/16 3y: 26.5/19.9 5y: 9.9/11.1	NA	NA	5/5 NA	NA	Retro	NA
Hasegawa [36]	117	55/62	2004–2016	63/66	45.1/64.2	60/88.7	c: 60/46.8	98.0/91.4	17.7–45.6/43.4 2y: 38.5–72.4/77.4	12.1–28.9 /25.5	89.1/45.2	1.8/1.6 5.5/1.6	40/29.0	Retro	94.5/88.7
Zhou [8]	282	187/95	2000–2019	61/65	NA	64.2/55.8	p: 55.1/72.0	75.4/74.7	11/18 NA	NA	52.9/66.3	7/0 18/4.2	NA	Retro PSM	89.3/90.5
Mangiameli [16]	163	78/85	2000–2021	60/65	47.4/57.6	53.8/88.2	p: 24.3/54.1	100/88.2	28.1/25.5 3y: 37.0/36.5 5y: 11.0/19.6	14.6/13.7	80.8/66.7	1.3/2.3 5.1/3.5	35.9/54.1	Retro	NA

*EPP* extrepleural pneumonectomy, *P/D* pleurectomy/decortication, *n* of Pts: number of patients, *MMT* multimodality treatment, *DFS* disease-free survival, *MCR* macroscopic complete resection, *(stage) c* clinical, *p* pathological, *NA* not available, *Retro* retrospective study, *PSM* propensity score matching

Regarding resection radicality, P/D is considered sensuously inferior to EPP [32]. On the other hand, reports state that the prognosis was extended by the advantage of adjuvant therapy and QoL [10, 11, 43]. However, because of the bias related to the subjects and uneven differences in MMT, the comparison between surgical procedures, which is retrospective, is meaningless. Therefore, both procedures having different therapeutic effects and characteristics should be considered incompatible. Yet, it is appropriate for an experienced surgeon to examine and select a wide range of surgical procedures according to each patient’s condition.

### Current status of P/D surgery

The term P/D was first proposed by Rusch in 1993 [30], but the technique has been revised by various institutions with varying styles, purposes, and nomenclature, and is currently not unified.

A consensus report jointly published by IASLC and IMIG in 2012 defined the term as follows [2]:

1. Extended P/D: parietal and visceral pleurectomy with resection of the diaphragm and/or pericardium and removal of all macroscopic tumors.
2. P/D: parietal and visceral pleurectomy with MCR without the diaphragm and/or pericardium resection.

These are the curative procedures, namely those that can achieve MCR. In terms of achieving MCR, the following questions have been raised:

1. If there is no tumor macroscopically, should the pleura be left intact, or should the organ pleura be completely resected because of the assumption of microscopic tumor?
2. If the tumor has invaded the lung parenchyma, should a combined resection of the lungs be performed, and should the procedure be evaluated differently depending on the extent of parenchymal resection?

In 2019, a joint effort arising from a task force formed at NCI-IASLC-MARF attempted to answer these questions and set out to form an international consensus on the nomenclature and description of surgical treatments for MPMs [22]. As a further improvement, a technique to perform P/D without touching the pleura where the tumor is involved was introduced in Japan, and our institution has been using this technique, whenever indicated, since 2020. We expect that this technique will contribute to improving the prognosis of patients with local recurrence and other life-threatening diseases (no-touch technique: non-incisional P/D) [38].

Finally, we present the perioperative results of P/D performed at our hospital. A total of 204 patients underwent P/D (median age, 67 years; range, 16–82 years). Men accounted for 80.9% (*n* = 165) of the patients. The histologic tumor types were epithelioid/biphasic/sarcomatoid (*n* = 188/13/4). IMIG pathological stage I (Ia + Ib) was confirmed in 54.9% (I/II/III/IV, *n* = 112/11/73/8). Table 2 summarizes the perioperative data, including adverse events and, prognosis, of the patients who underwent P/D at our hospital. The 30-day and 90-day mortality rates of the patients who underwent P/D were 0.5% (1/204) and 2.0% (4/204), respectively. There were no grade 4 or higher adverse events related to chemotherapy or radiation therapy. Grade 3 or higher adverse events occurred in 41 patients (20.1%). Reoperation was required for 12 patients (5.9%), and a long-term air leak, defined as an air leak lasting more than 7 days, occurred in 119 patients (58.3%). Most of these patients required pleurodesis and re-drainage. The median follow-up time after diagnosis for survivors was 28.7 months (range 1–106 months). The median overall survival (MST) and progression-free survival (PFS) times for all patients (*n* = 204) were 42 and 12 months, respectively.

**Table 2:** Perioperative results of pleurectomy/decortication performed at Hyogo Medical University

n = 204	
Operation time (min), median (range)	471 (241–882)
Blood loss (g), median (range)	1393 (310–7648)
MCR, *n* (%)	193 (95.0)
Completion of MMT^a^, *n* (%)	150 (73.5)
30-day mortality, *n* (%)	1 (0.5)
90-day mortality, *n* (%)	4 (2.0)
Patients with AEs (all Gr), *n* (%)	135 (66.2)
Prolonged air leakage (> 7 days), *n* (%)	119 (58.3)
Patients with Gr ≧ 3 AEs, *n* (%)	41 (20.1)
Reoperation, *n* (%)	12 (5.9)
ARDS/interstitial pneumonia, *n* (%)	9 (4.4)
Empyema	7 (3.4)
Heart failure/arrhythmia	4 (2.0)
Overall survival (mo)	
2-year (95% CI)	68.0 (60.9–74.1)
5-year (95% CI)	35.0 (26.7–43.5)
Median (mo)	42
Progression-free survival (mo)	
2-year (95% CI)	34.3 (27.7–41.0)
5-year (95% CI)	14.1 (9.1–20.3)
Median (mo)	12

*MMT* multimodality treatment

^a^Patients who underwent both neoadjuvant and adjuvant chemotherapy

## Conclusions

The essence of surgical resection for MPM is to achieve MCR, which is possible by performing a minimum resection rather than the simple alternative between EPP and P/D. Nevertheless, achieving a QoL that can allow adjuvant therapy and optional treatment for any recurrence is of the utmost importance.

In the 2020s, lung-sparing surgery is an important part of MMT. Moreover, the surgeon should select the optimal surgical procedure according to the patient’s condition and the degree of tumor invasion. We must also be responsible for improving the patient’s prognosis from a long-term perspective, including the indications for treatment of postoperative recurrence and metastasis.

## References

[1] Sugarbaker DJ (2006). Macroscopic complete resection: the goal of primary surgery in multimodality therapy for pleural mesothelioma. J Thorac Oncol.

[2] Rusch V, Baldini EH, Bueno R, De Perrot M, Flores R, Hasegawa S (2013). The role of surgical cytoreduction in the treatment of malignant pleural mesothelioma: meeting summary of the International Mesothelioma Interest Group Congress, September 11–14, 2012, Boston. Mass J Thorac Cardiovasc Surg.

[3] Krug LM, Pass HI, Rusch VW, Kindler HL, Sugarbaker DJ, Rosenzweig KE (2009). Multicenter phase II trial of neoadjuvant pemetrexed plus cisplatin followed by extrapleural pneumonectomy and radiation for malignant pleural mesothelioma. J Clin Oncol.

[4] Cao C, Tian D, Park J, Allan J, Pataky KA, Yan TD (2014). A systematic review and meta-analysis of surgical treatments for malignant pleural mesothelioma. Lung Cancer.

[5] Zalcman G, Mazieres J, Margery J, Greillier L, Audigier-Valette C, Moro-Sibilot D (2016). Bevacizumab for newly diagnosed pleural mesothelioma in the Mesothelioma Avastin Cisplatin Pemetrexed Study (MAPS): a randomised, controlled, open-label, phase 3 trial. Lancet.

[6] Scherpereel A, Opitz I, Berghmans T, Psallidas I, Glatzer M, Rigau D (2020). ERS/ESTS/EACTS/ESTRO guidelines for the management of malignant pleural mesothelioma. Eur Respir J.

[7] Opitz I, Scherpereel A, Berghmans T, Psallidas I, Glatzer M, Rigau D (2020). ERS/ESTS/EACTS/ESTRO guidelines for the management of malignant pleural mesothelioma. Eur J Cardiothorac Surg.

[8] Zhou N, Rice DC, Tsao AS, Lee PP, Haymaker CL, Corsini EM (2021). Extrapleural pneumonectomy versus pleurectomy/decortication for malignant pleural mesothelioma. Ann Thorac Surg.

[9] Kanayama M, Mori M, Matsumiya H, Taira A, Shinohara S, Takenaka M (2022). Surgical strategy for malignant pleural mesothelioma: the superiority of pleurectomy/decortication. Surg Today.

[10] Luckraz H, Rahman M, Patel N, Szafranek A, Gibbs AR, Butchart EG (2010). Three decades of experience in the surgical multi-modality management of pleural mesothelioma. Eur J Cardiothorac Surg.

[11] Lapidot M, Gill RR, Mazzola E, Freyaldenhoven S, Swanson SJ, Jaklitsch MT (2020). Pleurectomy decortication in the treatment of malignant pleural mesothelioma: encouraging results and novel prognostic implications based on experience in 355 consecutive patients. Ann Surg.

[12] Flores RM, Pass HI, Seshan VE, Dycoco J, Zakowski M, Carbone M (2008). Extrapleural pneumonectomy versus pleurectomy/decortication in the surgical management of malignant pleural mesothelioma: results in 663 patients. J Thorac Cardiovasc Surg.

[13] Burt BM, Cameron RB, Mollberg NM, Kosinski AS, Schipper PH, Shrager JB (2014). Malignant pleural mesothelioma and the Society of Thoracic Surgeons Database: an analysis of surgical morbidity and mortality. J Thorac Cardiovasc Surg.

[14] Batirel HF, Metintas M, Caglar HB, Ak G, Yumuk PF, Yildizeli B (2016). Adoption of pleurectomy and decortication for malignant mesothelioma leads to similar survival as extrapleural pneumonectomy. J Thorac Cardiovasc Surg.

[15] Sharkey AJ, Tenconi S, Nakas A, Waller DA (2016). The effects of an intentional transition from extrapleural pneumonectomy to extended pleurectomy/decortication. Eur J Cardiothorac Surg.

[16] Mangiameli G, Bottoni E, Cariboni U, Ferraroli GM, Morenghi E, Giudici VM (2022). Single-center 20-year experience in surgical treatment of malignant pleural mesothelioma. J Clin Med.

[17] Baas P, Scherpereel A, Nowak AK, Fujimoto N, Peters S, Tsao AS (2021). First-line nivolumab plus ipilimumab in unresectable malignant pleural mesothelioma (CheckMate 743): a multicentre, randomised, open-label, phase 3 trial. The Lancet.

[18] Chintala NK, Restle D, Quach H, Saini J, Bellis R, Offin M (2021). CAR T-cell therapy for pleural mesothelioma: rationale, preclinical development, and clinical trials. Lung Cancer.

[19] Nakamura A, Takuwa T, Hashimoto M, Kuroda A, Nakamichi T, Matsumoto S (2020). Clinical outcomes with recurrence after pleurectomy/decortication for malignant pleural mesothelioma. Ann Thorac Surg.

[20] Dawson AG, Kutywayo K, Mohammed SB, Fennell DA, Nakas A (2022). Cytoreductive surgery with hyperthermic intrathoracic chemotherapy for malignant pleural mesothelioma: a systematic review. Thorax.

[21] Cameron RB (2007). Extrapleural pneumonectomy is the preferred surgical management in the multimodality therapy of pleural mesothelioma: con argument. Ann Surg Oncol.

[22] Friedberg JS, Culligan MJ, Tsao AS, Rusch V, Sepesi B, Pass HI (2019). A proposed system toward standardizing surgical-based treatments for malignant pleural mesothelioma, from the joint national cancer institute-international association for the study of lung cancer-mesothelioma applied research foundation taskforce. J Thorac Oncol.

[23] Treasure T, Lang-Lazdunski L, Waller D, Bliss JM, Tan C, Entwisle J (2011). Extra-pleural pneumonectomy versus no extra-pleural pneumonectomy for patients with malignant pleural mesothelioma: clinical outcomes of the Mesothelioma and Radical Surgery (MARS) randomised feasibility study. Lancet Oncol.

[24] Kantor T, Wakeam E (2021). Landmark trials in the surgical management of mesothelioma. Ann Surg Oncol.

[25] Weder W, Stahel RA, Baas P, Dafni U, de Perrot M, McCaughan BC (2011). The MARS feasibility trial: conclusions not supported by data. Lancet Oncol.

[26] Waller DA, Opitz I, Bueno R, Van Schil P, Cardillo G, Harpole D (2021). Divided by an ocean of water but united in an ocean of uncertainty: a transatlantic review of mesothelioma surgery guidelines. J Thorac Cardiovasc Surg.

[27] Butchart EG, Ashcroft T, Barnsley WC, Holden MP (1976). Pleuropneumonectomy in the management of diffuse mailgnant mesothelioma of the pleura. Experience with 29 patients. Thorax.

[28] McCormack PM, Nagasaki F, Hilaris BS, Martini N (1982). Surgical treatment of pleural mesothelioma. J Thorac Cardiovasc Surg.

[29] Martini N, Bains MS, Beattie EJ (1975). Indications for pleurectomy in malignant effusion. Cancer.

[30] Rusch VW (1993). Pleurectomy/decortication and adjuvant therapy for malignant mesothelioma. Chest.

[31] Rusch VW (2012). Extrapleural pneumonectomy and extended pleurectomy/decortication for malignant pleural mesothelioma: the Memorial Sloan-Kettering Cancer Center approach. Ann Cardiothorac Surg.

[32] Friedberg JS (2013). The state of the art in the technical performance of lung-sparing operations for malignant pleural mesothelioma. Semin Thorac Cardiovasc Surg.

[33] Hasegawa S (2014). Extrapleural pneumonectomy or pleurectomy/decortication for malignant pleural mesothelioma. Gen Thorac Cardiovasc Surg.

[34] Okada M, Mimura T, Ohbayashi C, Sakuma T, Soejima T, Tsubota N (2008). Radical surgery for malignant pleural mesothelioma: results and prognosis. Interact Cardiovasc Thorac Surg.

[35] Lang-Lazdunski L, Bille A, Lal R, Cane P, McLean E, Landau D (2012). Pleurectomy/decortication is superior to extrapleural pneumonectomy in the multimodality management of patients with malignant pleural mesothelioma. J Thorac Oncol.

[36] Hasegawa S, Kondo N, Matsumoto S, Takuwa T, Hashimoto M, Kuroda A (2019). Surgical risk and survival associated with less invasive surgery for malignant pleural mesothelioma. Semin Thorac Cardiovasc Surg.

[37] Hasegawa S, Yokoi K, Okada M, Tanaka F, Shimokawa M, Daimon T (2021). Neoadjuvant pemetrexed plus cisplatin followed by pleurectomy for malignant pleural mesothelioma. J Thorac Cardiovasc Surg.

[38] Hasegawa S, Hashimoto M, Kondo N, Miyamoto Y (2020). Non-incisional pleurectomy/decortication. Eur J Cardiothorac Surg.

[39] Hasegawa S, Okada M, Tanaka F, Yamanaka T, Soejima T, Kamikonya N (2016). Trimodality strategy for treating malignant pleural mesothelioma: results of a feasibility study of induction pemetrexed plus cisplatin followed by extrapleural pneumonectomy and postoperative hemithoracic radiation (Japan Mesothelioma Interest Group 0601 Trial). Int J Clin Oncol.

[40] Rusch VW, Giroux D, Kennedy C, Ruffini E, Cangir AK, Rice D (2012). Initial analysis of the international association for the study of lung cancer mesothelioma database. J Thorac Oncol.

[41] Aziz T, Jilaihawi A, Prakash D (2002). The management of malignant pleural mesothelioma; single centre experience in 10 years. Eur J Cardiothorac Surg.

[42] Cao C, Tian D, Manganas C, Matthews P, Yan TD (2012). Systematic review of trimodality therapy for patients with malignant pleural mesothelioma. Ann Cardiothorac Surg.

[43] Raskin J, Surmont V, Cornelissen R, Baas P, van Schil PEY, van Meerbeeck JP (2018). A randomized phase II study of pleurectomy/decortication preceded or followed by (neo-)adjuvant chemotherapy in patients with early stage malignant pleural mesothelioma (EORTC 1205). Transl Lung Cancer Res.

[44] Scherpereel A, Astoul P, Baas P, Berghmans T, Clayson H, de Vuyst P (2010). Guidelines of the European Respiratory Society and the European Society of Thoracic Surgeons for the management of malignant pleural mesothelioma. Eur Respir J.

[45] Klotz LV, Hoffmann H, Shah R, Eichhorn F, Gruenewald C, Bulut EL (2022). Multimodal therapy of epithelioid pleural mesothelioma: improved survival by changing the surgical treatment approach. Transl Lung Cancer Res.

[46] Chan WH, Sugarbaker DJ, Burt BM (2017). Intraoperative adjuncts for malignant pleural mesothelioma. Transl Lung Cancer Res.

[47] Friedberg JS, Culligan MJ, Mick R, Stevenson J, Hahn SM, Sterman D (2012). Radical pleurectomy and intraoperative photodynamic therapy for malignant pleural mesothelioma. Ann Thorac Surg.

[48] Nakamichi T, Hashimoto M, Nakamura A, Kuroda A, Tanaka T, Takeuchi J (2021). Quality of life and lung function after pleurectomy/decortication for malignant pleural mesothelioma. Interact Cardiovasc Thorac Surg.

[49] Schwartz RM, Lieberman-Cribbin W, Wolf A, Flores RM, Taioli E (2018). Systematic review of quality of life following pleurectomy decortication and extrapleural pneumonectomy for malignant pleural mesothelioma. BMC Cancer.

[50] Ploenes T, Osei-Agyemang T, Krohn A, Waller CF, Duncker-Rohr V, Elze M (2013). Changes in lung function after surgery for mesothelioma. Asian Cardiovasc Thorac Ann.

[51] Takuwa T, Hashimoto M, Matsumoto S, Kondo N, Kuribayash K, Nakano T (2017). Post-recurrence chemotherapy for mesothelioma patients undergoing extrapleural pneumonectomy. Int J Clin Oncol.

[52] Lim E, Darlison L, Edwards J, Elliott D, Fennell DA, Popat S (2020). Mesothelioma and Radical Surgery 2 (MARS 2): protocol for a multicentre randomised trial comparing (extended) pleurectomy decortication versus no (extended) pleurectomy decortication for patients with malignant pleural mesothelioma. BMJ Open.

[53] Bovolato P, Casadio C, Bille A, Ardissone F, Santambrogio L, Ratto GB (2014). Does surgery improve survival of patients with malignant pleural mesothelioma?: a multicenter retrospective analysis of 1365 consecutive patients. J Thorac Oncol.

[54] Kostron A, Friess M, Inci I, Hillinger S, Schneiter D, Gelpke H (2017). Propensity matched comparison of extrapleural pneumonectomy and pleurectomy/decortication for mesothelioma patients. Interact Cardiovasc Thorac Surg.

[55] Verma V, Ahern CA, Berlind CG, Lindsay WD, Sharma S, Shabason J (2017). National cancer database report on pneumonectomy versus lung-sparing surgery for malignant pleural mesothelioma. J Thorac Oncol.

[56] Arifin AJ, Al-Shafa F, Chen H, Boldt RG, Warner A, Rodrigues GB (2020). Is lung stereotactic ablative radiotherapy safe after pneumonectomy?—a systematic review. Transl Lung Cancer Res.

[57] Cao CQ, Yan TD, Bannon PG, McCaughan BC (2010). A systematic review of extrapleural pneumonectomy for malignant pleural mesothelioma. J Thorac Oncol.

[58] Sugarbaker DJ, Richards WG, Bueno R (2014). Extrapleural pneumonectomy in the treatment of epithelioid malignant pleural mesothelioma: novel prognostic implications of combined N1 and N2 nodal involvement based on experience in 529 patients. Ann Surg.

